# Free Standing Dry and Stable Nanoporous Polymer Films Made through Mechanical Deformation

**DOI:** 10.1002/advs.202207472

**Published:** 2023-04-25

**Authors:** Hsiao‐Ping Hsu, Manjesh K. Singh, Yu Cang, Héloïse Thérien‐Aubin, Markus Mezger, Rüdiger Berger, Ingo Lieberwirth, George Fytas, Kurt Kremer

**Affiliations:** ^1^ Max‐Planck‐Institut für Polymerforschung Ackermannweg 10 55128 Mainz Germany; ^2^ Present address: Department of Mechanical Engineering IIT Kanpur Kanpur Uttar Pradesh 208016 India; ^3^ Present address: School of Aerospace Engineering and Applied Mechanics Tongji University Zhangwu Road 100 Shanghai 200092 China; ^4^ Present address: Chemistry Department Memorial University of Newfoundland St. John's Newfoundland Canada; ^5^ Present address: Faculty of Physics University of Vienna Boltzmanngasse 5 Wien 1090 Austria

**Keywords:** free‐standing polymer film, mechanical deformation, polymer glass, porosity

## Abstract

A new straight forward approach to create nanoporous polymer membranes with well defined average pore diameters is presented. The method is based on fast mechanical deformation of highly entangled polymer films at high temperatures and a subsequent quench far below the glass transition temperature *T*_g_. The process is first designed generally by simulation and then verified for the example of polystyrene films. The methodology does not need any chemical processing, supporting substrate, or self assembly process and is solely based on polymer inherent entanglement effects. Pore diameters are of the order of ten polymer reptation tube diameters. The resulting membranes are stable over months at ambient conditions and display remarkable elastic properties.

## Graphical abstract

Free standing, stable nanoporous membranes made of highly entangled polymers are created solely by applying a biaxial deformation at high temperature and a subsequent quench below the glass transition temperature. Porous structures, as observed from simulation and experiment, are governed by polymer chains extending beyond the pore diameters and display remarkable new elastic properties.

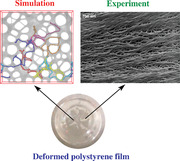

## 1 Introduction

Advanced water treatment, gas separation, medical, or food technologies are a few examples of applications in modern filtration processes, requiring well defined barrier materials with good control of their nano‐ or micro‐porous structure. Besides separation applications, specific large absorption areas or microscopically small reaction volumes offer unique new scientific and technological possibilities due to extremely high surface to volume ratios in such systems. Thus, it is not at all surprising that nano‐ and micro‐porous materials have become an important class of functional systems, which serve many purposes in our modern technology dominated world. There are several reviews on nano‐porous systems describing the above mentioned requirements in detail as well as application aspects^[^ ^1^, ^2^ ^]^ and prospective developments.^[^ ^3^ ^]^ A widely employed approach to create such systems uses block‐copolymers (for a very general introduction see ref. [4]), where ordering into columnar or double gyroid structures and subsequent removal of one kind of block or of solvent leads to well defined pore structures.^[^ ^1^, ^2^, ^5^, ^6^, ^7^, ^8^ ^]^ For that process membranes typically are supported by a substrate. In ref. [9] each block itself is a bottle brush polymer, allowing for larger pores. Besides synthetic copolymers also silk protein like tri‐block copolymers have been used.^[^ ^10^, ^11^ ^]^ Another self assembly and removal strategy is applied for metal organic frameworks (MOFs), where—in simple terms—one block is replaced by an inorganic compound.^[^ ^12^ ^]^ The resulting inorganic membrane can sustain much higher temperatures and often also mechanical load compared to organic polymer systems. All these processes are based on phase segregation of different blocks/components. The opposite, namely local solvation into small solution droplets and subsequent evaporation leading to so called breath figures, is used to create well defined patterns of small holes, employed especially for lithography purposes.^[^ ^13^ ^]^ Furthermore fast evaporation and polymer solvent phase separation can lead to hollow porous capsules^[^ ^14^ ^]^ or hollow porous fibers.^[^ ^15^ ^]^ Yet another more recent class of systems, which needs extreme precision treatment, is based on nano porous graphene membranes.^[^ ^16^, ^17^ ^]^ All these approaches require next to a substrate, high precision chemistry and highly controlled processing.

Alternatively, polymer based approaches not requiring such a precision chemistry, employ semiflexible filaments or network/gel structures. Swelling polymer gels or adding nanoscopic inclusions and subsequent fixation of the swollen network structure have been used to create microporous polymer systems.^[^ ^18^, ^19^ ^]^ For such gels both chemical connectivity as well as entanglements, that is, non‐crossability of the chains, determine structure and stability, as also known from early simulations.^[^ ^20^ ^]^ Related to that is a computer simulation based proposal to quench initially totally disordered solutions of rod like polymers very fast deeply into the two phase region, producing a morphology reminding of the molecular equivalent of a sticky stick Mikado game.^[^ ^21^ ^]^ Membranes for textiles, like GoreTex can be made by fast evaporation of solvent of deformed polymer solution films,^[^ ^22^ ^]^ however, with typical pore sizes of and above 500 nm.

This cursory list reveals the great scientific and technological demand of nanoporous systems. However, despite all progress, making them is rather complex, typically involving several components and precisely controlled processing steps, with thermal or chemical removal of at least one component. Furthermore, usually a solid or liquid support is required, making it highly desirable to find a simpler, more direct route to nanoporous films.^[^ ^3^ ^]^ One such simplification could be based but not restricted to commodity homopolymers. Here we present such an uncomplicated approach to create free standing, stable nanoporous films.

## 2 Concept and Realization

Some of the above mentioned works already required conserved entanglements. Here we go significantly beyond that and employ them to create nanoporous membranes. We start with highly entangled homopolymer melts, needing neither cross linking nor any phase segregation mechanism. Melt polymer chains assume Gaussian random walk structure with chain extension ∝*N* ^1/2^, *N* being the number of monomers per chain. Consequently, *O*(*N* ^1/2^) chains share the volume of a given chain, creating a high average density of transient, but long living topological constraints, entanglements, because chains cannot cut through each other. This leads above a characteristic chain length, the entanglement length *N*_e_ or molecular weight *M*_e_, to dynamics following the tube/reptation model,^[^ ^23^ ^]^ well supported by both experiment and simulation.^[^ ^24^, ^25^, ^26^ ^]^ These constraints cause very slow dynamics, that is, relaxation times τ_d_ ≈ *O*(*N* ^3.4^), which together with the highly temperature dependent bead friction easily become accessible experimentally or during processing.^[^ ^27^ ^]^ Here we take advantage of this interplay of slow dynamics and topological constraints and produce nanoporous polymer films just by a biaxial deformation well above the glass transition temperature and subsequent stabilization of this structure by a quench into the glassy state. Furthermore, we use melts of long chains of many entanglement lengths, which have the advantage of avoiding high chain end densities, which have been shown to facilitate instabilities and cracks under mechanical load.^[^ ^28^ ^]^ It should be mentioned there have been previous attempts to prepare microporous membranes by mechanical deformation as reported in several patents.^[^ ^29^, ^30^, ^31^, ^32^ ^]^ In the first work from 1988 deformation was used to introduce small crazes by a variety of deformation methods, which then had to be stabilized^[^ ^29^ ^]^ by further treatment. The authors assume micron sized pores, but did not provide further details. In other approaches ultra high molecular weight PE (UHW‐PE) of molecular weights of up to 12 × 10^6^ Da have been deformed.^[^ ^30^, ^31^, ^32^ ^]^ There pores from about 500 nm and above have been reported, where the membranes require special stabilization through for example, a rigid frame. Here we take a more microscopic view and do not need UHW polymers.

### 2.1 Porous Films Predicted by Modeling

We begin with thick fully equilibrated free standing polymer melt films of long bead spring chains, that is, *N* ≈ 72*N*_e_ with *N*_e_ = 28 for simulations and *M* ≈ 60*M*_e_ for experiment (see below), respectively. Typically a simulated film contains *n*_c_ = 1000 polymer chains of *N* = 2000 monomers. The initial thickness of the films is *h* ≈ 130σ along the *z*‐direction of the simulation box, leading to a lateral extension of *L*_*x*_ = *L*_*y*_ ≈ 134σ with periodic boundary conditions at the standard melt density of ρ = 0.85σ^−3^ and (simulation) pressure *P*_*zz*_ = 0ϵ/σ^3^. Units for simulation time (τ), length (σ), and energy (ϵ) are standard Lennard Jones units. The simulation temperature *k*_B_ *T* = 1ϵ, while the glass transition temperature *T*_g_ for this system is about *T*_g_ ≈ 0.67ϵ/*k*_B_,^[^ ^33^, ^34^ ^]^ which is the same as the bulk value. Details of the simulation model and deformation procedure are given in the Supporting Information.

**Figure 1:**
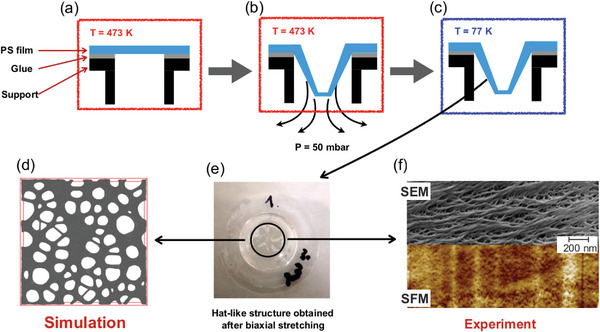
Porous film fabrication: We use monodisperse (polydispersity index *M*_W_/*M*_N_ = 1.04) polystyrene (PS) of molecular weight *M*_n_ = 1000 kDa (see Supporting Information). For PS^[^ ^36^ ^]^ *M*_e_ = 16.6 kDa corresponding to about *M*_n_ ≈ 60*M*_e_. Well equilibrated transparent PS films of about 100 µm thickness were prepared by pressing PS (250 mg) under 20 kN load and temperature 433 K, well above the glass transition temperature *T*_g_ = 380 K,^[^ ^37^ ^]^ for an hour. To biaxially deform, the PS film was immobilized by the edges over circular aperture of 1.75 cm diameter and deformed by applying a load using the negative pressure generated by a controlled vacuum of 50 mbar. This was achieved by attaching the film to one end of a vacuum flange using a cyanoacrylate glue. The flange was put in an oven and maintained at a temperature of 473 K. After 2 min, a vacuum of ≈50 mbar was applied for different durations. a–c) Schematic representation of experimental protocol to deform PS films into “hat” like shape using vacuum. a) Pre‐heating of polymer film at 473 K, b) applying a pressure of 50 mbar to deform the film into a “hat‐like” shape, and c) the deformed film is fast quenched to 77 K in a liquid nitrogen bath, d) applying an analogous protocol (a–c) in MD simulations to produce nanoporous films (top view), e) the hat as it is obtained from experiments after stretching and temperature quench, and f) SEM and SFM image of the same region of the top part of a hat (side view) of a P‐40 film (Table S1, Supporting Information). The porosity in the sample is clearly visible as also predicted by MD simulations. The black ink marks on the hat in (e) are for sample‐reference.

**Figure 2:**
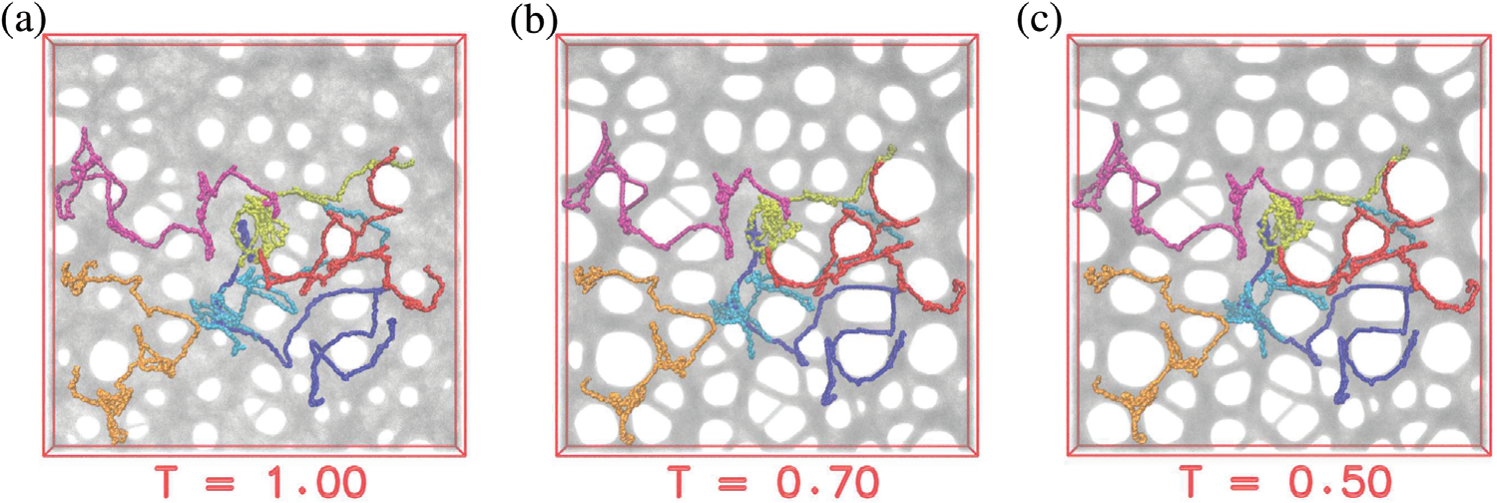
Porous film morphology. Simulated expanded films at *T*[ϵ/*k*_B_] = a)1.0, b) 0.7, and c) 0.5, with six marked randomly selected chains. The chains' extension is significantly larger than the pore diameter.

Films are quasi‐continuously expanded biaxially in the *x*‐ and *y*‐directions up to a strain of 4 × 4, while *h* freely adjusts. For most simulations we apply a strain rate of $\dot{\varepsilon }=77/\tau_{R}=0.015/\tau_{e}$. This is significantly faster than the Rouse relaxation rate of the whole chains, $\tau_{R}^{-1}\approx {\left(\tau_{0}N^{2}\right)}^{-1}$ but much slower than that of a subchain of an entanglement length, $\tau_{e}^{-1}\approx {\left(\tau_{0}N_{e}^{2}\right)}^{-1}$, that is, $\tau_{R}^{-1}<\dot{\varepsilon }<\tau_{e}^{-1}$. This choice ensures throughout the whole deformation process that chain conformations globally follow the deformation, while on small scales of the order of the tube diameter $d_{T}\propto N_{e}^{1/2}\sigma$ they can relax.^[^ ^23^, ^25^, ^35^ ^]^ The latter causes local stresses in chains to remain small, avoiding strain induced chain breakage (if compared to experiment). After deformation *L*_*x*, *y*_ ≈ 526σ and *h*(*t* = 0) ≈ 10.02σ initially. Upon subsequent relaxation and cooling below *T*_g_ at fixed strain *h* somewhat increases to 11.7σ. Below *T*_g_ no further morphology change is observed. This process reduces the global film density to ρ = 0.62σ^−3^ compared to ρ = 0.85σ^−3^ for the melt. The competition of very slow large scale chain relaxation and surface minimizing surface tension produces stable nanoporous structures, **Figures** **1**d and **2**, instead of an open network of strands or a single big hole. Chains extend beyond several pores, forming a stable, highly entangled network. The average pore diameter *D*_pore_ ≈ 40σ about eight times the equilibrium tube diameter *d*_T_ ≈ 5.02σ.^[^ ^26^ ^]^ From Figure 2 it becomes obvious that long chains extending over several holes are needed to stabilize the porous structure and that, as mentioned in the introduction, short chains could destabilize the films. Therefore we tested this with an example of polydispersity *M*_W_/*M*_N_ = 1.8. However, this level of polydispersity was not enough to destroy the porous structure (for details see Supporting Information). Furthermore, the image illustrates that occasional crosslinks along the backbone of the chains will not alter the outcome significantly, while a high crosslinking density creating a dense network could do that. We also tested the case of a somewhat faster deformation, within the strain rate constraints mentioned above, without any significantly different outcome.

This simulation protocol is easily transferred to experiment and suggests a direct way to create stable and well controlled porous (commodity) polymer membranes solely based on mechanical deformation and subsequent fast (compared to the chain relaxation time) cooling below *T*_g_.

### 2.2 Porous Films, Experimental Realization

Following the computer simulations, we prepare such porous films from standard commodity polymers as described in Figure 1. The simulation protocol does not require any specific chemistry up to the condition of highly entangled long chain polymer systems, which at ambient temperature (or more generally at the temperature of experimental tests or practical use) are well below the glass transition temperature. Thus, without any loss of generality we use monodisperse (polydispersity index *M*_W_/*M*_N_=1.04) polystyrene (PS) films of molecular weight *M*_n_ = 1000 kDa with a thickness of about 100 µm. For PS^[^ ^36^ ^]^ *M*_e_ = 16.6 kDa, which corresponds to about *M*_n_ ≈ 60*M*_e_ and a reptation tube diameter *d*_T, PS_ ≈ 5 nm. These films were biaxially expanded well above *T*_g_. To preserve the morphology of the stretched films, they are quenched far below *T*_g_ by dipping them into liquid nitrogen as illustrated in Figure 1a–c and as detailed in the Supporting Information. The resulting hat like structures (Figure 1e) were stored at room temperature, without taking any further precautions. Depending on the duration of vacuum pressure applied, different degrees of stretching were achieved in PS films as given in Table S1, Supporting Information. The time scales involved are significantly larger than simulation times.

This process leads to films of average thickness h≅12µm in the presumably most expanded region on the top of the “hats” (Figure 1e) in P‐40 films (see Table S1, Supporting Information) and estimated expansion ratio of λ_L_ close to ≈3.5 in that region. Resulting densities were determined from sample mass and volume. Alternatively, measuring gas adsorption leads to 43% porosity (Figure S4, Supporting Information) in P‐40 films. From that we expect the structural properties of the experimental systems to roughly resemble those predicted by simulations. Figure 1f shows a typical electron micrograph (SEM) and a scanning force measurement (SFM) image of a P‐40 film, indicating that we indeed obtain a nanoporous morphology. To illustrate that the observed morphology is not an artefact of the fracture process used for the SEM micrograph, we also compare this to the unperturbed film, as shown in Figure S6, Supporting Information.

We have prepared several groups of porous films following protocols indicated in Table S1, Supporting Information. The film porosity strongly correlates with the stretching protocol. Here, we present results for four different groups of porous films as listed in **Table** **1**, and Table S1, Supporting Information. Henceforth, the samples will be referred to as P‐0, P‐10, P‐26, and P‐40.

**Table 1:** Film density ρ, effective refractive index *n*_eff_, and porosity ϕ ($\phi^{\#}$ estimated according to $n_{\text{eff}}^{2}=\phi^{\#}n_{\text{air}}^{2}+\left(1-\phi^{\#}\right)n_{\text{PS}}^{2}$ and ϕ* obtained from BET measurement (Figure S4, Supporting Information) of examined porous and bulk PS films

Sample	Density	Effective refractive	Porosity $\phi^{\#}$	Porosity ϕ*
ID	ρ [g cm^−3^]	index *n*_eff_		
P‐0	0.98	1.596	0	
P‐10	0.94	1.545	0.10	
P‐26	0.83	1.464	0.26	
P‐40	0.74	1.385	0.40	0.43

## 3 Porous Film Properties—Experiment and Simulation

Crucial to both is that expansion time scales fall well in between global and local chain relaxation times. During and right at the end of the expansion overall affinely deformed polymer conformations would be anticipated,^[^ ^38^ ^]^ conserving topological constraints, that is the tube structure. The entanglement molecular weight (*M*_e_, *N*_e_) and the resulting tube diameters define the length scale on which the polymer motion in equilibrium is confined to follow the reptation tube, which follows the overall coarse grained contour of the chain. *d*_T_ typically is taken to be  2^1/2^ the radius of gyration of a subchain of length *N*_e_ or *M*_e_. Up to that scale monomers experience the other surrounding chains essentially as a viscous background (i.e., the Rouse model), while beyond that topological constraints prevent the isotropic motion. For all amorphous polymers the length scale *d*_T_ and the related time scale τ_e_, which is the Rouse time of a subchain of length *N*_e_ or *M*_e_, depend on chemical or (for simulations) model details. However, normalized by these quantities viscoleastic properties of amorphous polymer melts follow the same universal bahavior.^[^ ^23^, ^39^ ^]^ Consequently, for matching experiment and simulation the respective entanglement molecular weights and resulting tube diameters define the length scales. With *d*_T, sim_ ≈ 5.0σ and *d*_T, PS_ ≈ 5.0 nm^[^ ^36^ ^]^ a length of 1σ in simulations corresponds to about 1 nm in experiment. For details of the determination of *d*_T_ and the comparison see Supporting Information. Hence with the estimated maximal film expansion in experiment of about 3.5 × 3.5 a semi quantitative comparison to the simulations can be expected.

Figure 1d,f allows for first visual comparisons. Certainly, pores are polydisperse with different spatial orientations. Keeping this in mind, in simulations average pore diameters *d*_p_ ≈ 40σ ≈ 8*d*_T_ are observed with no change below *T*_g_. Already ≈20% above *T*_g_ the bead friction becomes so large rendering observable changes very small, compared with Figure 2. In comparison, Figure 1f (and more in the Supporting Information) shows an electron micrograph (SEM) and a scanning force measurement (SFM) image of a P‐40 film. A rough analysis suggests pore diameters between ≈20 nm and ≈50 nm, that is, 4 to 10 *d*_T_, close to the expectation from simulation, even though experimental annealing and cooling times are much longer (see also Supporting Information). The tube diameter defines a typical effective mesh size,^[^ ^40^ ^]^ which also is critical for the pore diameter. In earlier work we have seen^[^ ^41^ ^]^ that the topological constraints can somewhat slide along each other upon uniaxial strain, but then jam and form rather tightly knotted, longer living regions. Finding pores of several tube diameters, but not more, nicely agrees with these earlier findings. By the fast temperature quench below *T*_g_ this non‐equilibrium morphology is stabilized.

The porosity ϕ from simulations is estimated by the ratio of the sub‐volume *V*_void_ accessible for a test particle of diameter 1σ^[^ ^42^, ^43^ ^]^ and the total system volume *V*_film_ = *hL*_*x*_ *L*_*y*_, namely $\phi =\frac{V_{\mathrm{void}}}{V_{\mathrm{film}}}\times 100\%$ (see Supporting Information). We find ϕ≈41% in good agreement with our earlier estimate based on the global density. Experimentally an estimate of ϕ provided by *N*_2_ gas adsorption experiments at 77 K, applying the BET model^[^ ^44^ ^]^ gives a pore volume of about 0.552 cm^3^ g^−1^ compared to 0.008 cm^3^ g^−1^ for the native film, suggesting ϕ_exp_ between 40% and 50% of the original film Table 1, for details see Supporting Information.

### 3.1 Scattering Function

While the visual inspection and pore size analysis of the porous films provides evidence for good general agreement, a detailed comparison of scattering functions yields more insight into the internal structure of the porous systems, by that providing a solid basis for structural comparison. To avoid artefacts from film surfaces and different film thicknesses in comparing scattering functions from simulation and experiment we restrict ourselves to in‐expansion‐plane isotropically averaged structure factors.

For simulations the form factor for each monomer, structureless Lennard Jones particles, is taken as *F*(*q*) = 1, independent of *q*. The in plane structure factor of the films, *S*(*q*), with $q_{∥}=q_{x}\hat{x}+q_{y}\hat{y}=2\pi \left(n_{1},n_{2},0\right)/L_{x}$ with *n*_1_ , *n*_2_ = 0, ±1, ±2, … then reads:

(1) $$S\left(q=|q_{∥}|\right)=\frac{1}{n_{c}N}\langle \sum_{i=1}^{n_{c}N}\sum_{j=1}^{n_{c}N}\exp\left(iq_{∥}\cdot r_{ij}\right)\rangle$$

where **r**_*ij*_ = **r**_*j*_ − **r**_*i*_ is the vector between monomer *i* and monomer *j* in the film.

**Figure 3:**
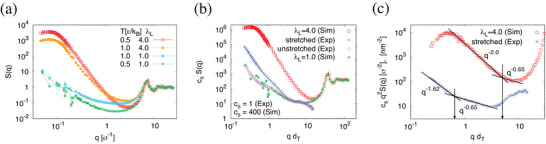
In‐expansion‐plane structure factor: a) Structure factor *S*(*q*) as a function of *q* for simulated expanded films at expansion rations of λ_L_ = 1.0 and 4.0 and two different temperatures *T*, as indicated (*q* > 3.5(2π/*L*_*x*_) for λ_L_ = 4.0. b) Comparison of *S*(*q*) between experiments (sample P‐40 at *T* = 295 K) and simulations (at *T* = 0.5ϵ/*k*_B_) roughly in the same supercooled regime (20% below *T*_g_): *c*_s_ *S*(*q*), plotted versus *qd*_T_, where the scaling factor *c*_*s*_ is determined such that the simulated *S*(*q*) for unstretched film is within error on top of the experimental data. c) Kratky plot *q* ^2^ *S*(*q*) multiplied by *c*_s_ of the stretched films of (b). The simulation *S*(*q*) shows very good agreement to the Porod law *S*(*q*)∝*q* ^−4^ at intermediate *q* values, while the fit for experiment gives *q* ^−3.62^. The crossover points between two linear lines at *qd*_T_ ≈ 0.65 (Exp) and 5.0 (Sim) correspond to 48 nm for the experimental, and 6.3σ for the simulation data. The latter is based on the assumption of the same sequence of power laws as in experiment.

**Figure** **3**a shows *S*(*q*) from simulation data for the unperturbed (λ_L_ = 1) and the most expanded state (λ_L_ ≈ 4) at *T* = 1ϵ/*k*_B_ right after the deformation and quenched to *T* = 0.5ϵ/*k*_B_, deep in arrested glassy state. Other stretching ratios are shown in Figure S1, Supporting Information. On short distances *d* ⩽ 3σ, that is, *q* > 2π/*d* = 2σ^−1^, the melt structure remains essentially unchanged with an amorphous halo around *q* = 6.9σ^−1^, which is a bit more pronounced, but not shifted for *T* = 0.5ϵ/*k*_B_. Thus the deformation process does not alter the local bead packing, as expected from the applied strain rates. On intermediate larger distances up to several tube diameters *S*(*q*) of the unstretched system indicates slightly larger fluctuations at high temperature compared to the low temperature for the undeformed film. Toward even smaller *q* these deviations vanish, as one would expect for an overall homogeneous system. For expanded films a sharp increase of *S*(*q*) compared to the unperturbed melt occurs, indicating large spatial inhomogeneities, with only minor differences between the high and low *T* data. This strong increase of *S*(*q*) for expanded films levels off in a broad maximum/shoulder below *q* = 0.1σ^−1^, a *q*‐value closely corresponding to the upper limit of pore diameters observed in simulations. The pores and their spatial distribution collectively lead to the large inhomogeneities in the expanded materials. For the stable, glassy system (*T* = 0.5ϵ/*k*_B_) this effect is even a bit more pronounced due to some hole coarsening and pore interface sharpening. However, much larger simulation systems would be needed to safely extrapolate *q* → 0 to estimate the compressibility from *S*(*q*).

To compare scattering data from experiment and simulation we take advantage of the length scaling, namely the normalization of lengths by the respective reptation tube diameters. Therefore we plot *S*(*q*) versus the dimensionless scattering vector *qd*_T_ in the subsequent analysis. Unscaled experimental data are given in Figures S8 and S9, Supporting Information. Small angle X‐ray scattering (SAXS) data of glassy native and expanded systems from simulation (*T* = 0.5ϵ/*k*_B_) and experiment (*T* ≈ 295 K) for the same in‐expansion scattering plane are shown in Figure 3b. In both cases $T\approx \frac{3}{4}T_{g}$. The only adjustable parameter is a vertical shift of *S*(*q*) as SAXS intensities are in arbitrary units. A first striking result is the perfect agreement of *S*(*q*) of native PS and of simulated films for nearly two decades, that is, a range from the experimentally accessible 128 nm down to about 3 nm (i.e., *qd*_T_ ≈ 10). For even smaller distances local packing differences originating from chemical details of PS and structureless simulated particles and from the form factor *F*(*q*) become relevant and lead to expected deviations.

For expanded systems, qualitative agreement between *S*(*q*) from experiment and simulations remains. First, as for simulations also in experiment we observe that the local monomer packing on distances below about 1.5*d*_T_ remains essentially unchanged. This confirms our assumption that the deformation process also in experiment is too slow to affect the local melt structure. For smaller *q*, just as in simulation, *S*(*q*) increases steeply due to inhomogeneities in the expanded films. However the broad low *q* plateau found in simulations is not yet reached experimentally, indicating that even lower *q* values would be required (this is in qualitative agreement to a rough pore spacing estimate from Brillouin scattering, see Figure S13, Supporting Information). Considering simulation results and the SEM and SFM images one would expect a similar broad plateau in experimental *S*(*q*) just below the smallest experimentally accessible *q* (Figure 3b). Furthermore the weaker increase of *S*(*q*) points toward a somewhat smaller expansion ratio, again in agreement with estimates given above. Despite this quantitative deviation the two cases excellently agree qualitatively, as is more clearly shown in the Kratky plot of *S*(*q*), Figure 3c (see also Figure S9, Supporting Information). At low *q*, *S*(*q*) follows a power law close to Porod's law, *S*(*q*)∝*q* ^−4^, crossing over to *q* ^−2.65^ for larger *q*, the latter more clearly expressed in experiment. These power laws are considered to be characteristic for nanoporous systems.^[^ ^45^, ^46^, ^47^ ^]^ The Porod regime is associated with the presence of sharp interfaces. Thus the walls of the pores are well defined and do not show significant roughness beyond a microscopic scale. For PS films the power law seems slightly smaller (*q* ^−3.62^), indicating interfaces with slightly increased surface roughness.

At the crossover to the *q* ^−2.65^ regime (2π/*q* ≈ 48 nm (experiment) and 2π/*q* ≈ 6.3σ (estimated, simulations)) the local wall structure becomes dominant, as argued in the literature.^[^ ^48^ ^]^ While for simulations only a deviation from Porod's law is clearly observed, a *q* ^−2.65^ regime can be clearly identified experimentally. The latter agrees to the pore diameters observed by electron microscopy and is considered to be a pore diameter signature.^[^ ^49^ ^]^ For simulations no change of *S*(*q*) power law around the average pore diameter is observed. It remains to be seen, whether and to what extent local differences in the surface structure or of the overall expansion ratio can lead to such a deviation on length scales between average pore diameter and microscopic monomer packing. Some first simulation tests with lower expansion ratios indicate a slightly better agreement to experiment at this point, suggesting that this effect also might be a matter of film expansion. This, however, is beyond the scope of the present work.

### 3.2 Elasticity of the Porous Films

To summarize the results so far, we have demonstrated that nanoporous films, as predicted by simulation, can be made by a simple biaxial expansion process and subsequent quenching into the glassy state of a highly entangled polymer melt. Because *T*_g_ of PS is way above room temperature, the segmental mobility is mostly frozen and these films turn out to be stable for months at ambient conditions. As shown from the simulations chains are significantly stretched and extend over several pores, forming an entangled network. This frozen network prevents further coarsening and keeps the porous membranes stable. However, this chain stretching is expected to lead to local conformation‐induced interactions, which are not that easy to identify by X‐ray scattering and BET techniques. Instead, the film elastic moduli should be a sensitive index of chain stretching and interactions as both are strongly impacted by local chain packing.^[^ ^50^ ^]^ Brillouin light spectroscopy (BLS) is a non‐contact, non‐invasive, and zero‐strain method for the direct measurement of Young's elastic modulus^[^ ^50^ ^]^ and should yield insight into elastic mechanical properties of this new material.

**Figure 4:**
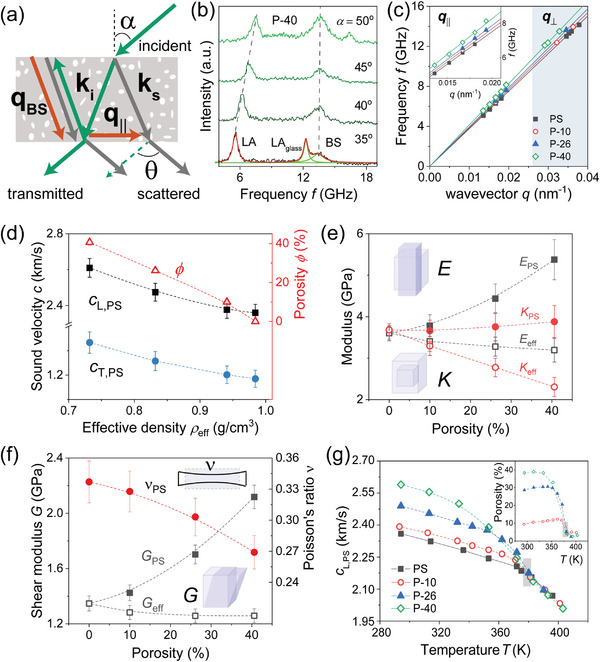
Thermoelasticity of the nanoporous PS films. a) Schematic of transmission geometry. The phonon wavevector **q**_∥_ = **k**_*s*_ − **k**_*i*_, **k**_*s*_ and **k**_*i*_ being wave vectors of the scattered and the incident light in the film directs in the film plane when the scattering angle θ is half of the incident angle α. Besides the inelastic scattering at *q*_∥_, the wavevector *q*_BS_ from the backscattered incident beam with **k**_*i*_ can be also selected. b) Exemplary polarized BLS spectra of the P‐40 film at different incident angles recorded with the scattering geometry of (a). The three peaks are assigned to the LA phonons with *q*_∥_ and *q*_BS_ denoted as BS in the porous film, and the longitudinal phonon with *q*_∥_ in the glass substrate. The spectrum is represented with three Lorentzians (green lines shown at α = 35^o^). c) Phonon dispersion relation for bulk and porous PS films recorded with *q*_∥_ and *q*_⊥_ normal to the film (shaded area). The regime recorded with *q*_∥_ is magnified in the inset. d) Sound velocity and porosity as a function of the film density. e) Young's (*E*) and bulk (*K*) moduli, and f) shear modulus for PS frame (subscript PS) and effective medium (subscript eff), and Poisson's ratio ν as a function of film porosity with the schematic of the corresponding elastic deformations definitions shown in the insets. g) Longitudinal sound velocity *c*_L, PS_(*T*) of bulk and porous PS films as a function of temperature. The shaded areas denote *T*_g_ of the bulk PS film as estimated from the kink of the linear fits (solid lines) of *c*_L, PS_(*T*). The corresponding porosity versus *T* is shown in the inset. All dashed lines are guides to the eye.

BLS is based on the inelastic scattering of light by propagating thermal phonons at frequencies shifted by ω = ±*cq* on both sides (Stoke, anti‐Stokes) of the elastic Rayleigh line (at ω = 0), where *c* is the sound velocity and *q* is the phonon wave vector selected by the scattering angle θ. In a homogeneous medium (*qd*_T_ < <1), the phonon dispersion ω(*q*) = *c*_L, *T*_ *q* is linear. Depending on the incident and scattered light polarization it yields the longitudinal *c*_L_ and transverse *c*_T_ (for solid materials) sound velocities, which leads to the longitudinal $M=\rho c_{L}^{2}$ and shear $G=\rho c_{T}^{2}$ moduli. Due to frequencies of BLS experiments (GHz range), moduli *M*, *G* present the elastic response of the materials. With the Poisson ratio ν = [((*M*/*G*)^2^ − 2)/((*M*/*G*)^2^ − 1)]/2 this also yields Young's modulus *E* = 2*G*(1 + ν). We utilize two scattering geometries allowing selection of *q* either parallel (**Figure** **4**a) or normal to the porous film (see Figure S10, Supporting Information). In the parallel configuration the magnitude *q*_∥_ = (4π/λ)sin α is independent of the refractive index *n*, whereas in the normal case, *q*_⊥_ = (4π/λ)(*n* ^2^ − sin ^2^α)^1/2^ depends on *n* with α being the incident angle and λ(= 532 nm) the laser wavelength in vacuum.^[^ ^51^ ^]^ Since the overall film is virtually isotropic on optical length scales, recording ω(*q*) along the two orthogonal directions enables an estimate of *n*(ϕ)^[^ ^52^ ^]^ and then the film porosity through the Maxwell–Garnett approximation $n^{2}\left(\phi \right)=n_{\mathrm{PS}}^{2}\left(1-\phi \right)+\phi n_{\mathrm{air}}^{2}$. where refractive indices *n*_air_(= 1) and *n*_PS_ = 1.596 are for air and bulk PS, respectively. For details see Supporting Information.

Figure 4 summarizes the BLS experiments. Typical polarized BLS spectra (anti‐Stokes) for a film (P‐40) with 40% porosity at four scattering angles (θ = 2α) with *q*_∥_ are shown in Figure 4b, represented by a Lorentzian line to yield the peak frequency *f*(*q*) for the longitudinal acoustic (LA) mode. The two additional peaks are due to the LA mode from backscattering (BS) and the LA of the glass substrate. For optically isotropic materials the dispersion *f*(*q*) encompassing *q*'s from both scattering configurations allows to estimate *n*. The LA phonon dispersion *f*(*q*) with *q*_∥_ and *q*_⊥_, as depicted in Figure 4c for three porous films is linear, yielding *c*_L_ = 2π*f*(*q*)/*q*, (2605 ± 40 m s^−1^) and *n* (=1.39 ± 0.03) for P‐40. From the depolarized BLS spectra, (see Figure S11, Supporting Information), the values of *c*_T_ = (1470 ± 50) m s^−1^ and the Poisson's ratio ν = 0.27 ± 0.01 are obtained for the same system. For comparison, Figure 4c includes phonon dispersion of the native PS film (ϕ = 0) with *M*_n_ = 1000 kDa giving *c*_L_ = 2360 ± 50 m s^−1^ and *n* = 1.59 ± 0.02. While *n*(ϕ) decreases with ϕ, phonons unexpectedly propagate faster in the nanoporous films than in the denser pristine PS (magnified inset to Figure 4c).

*c*_L_(ϕ) systematically increases with ϕ or equivalently decreases with increasing polymer density of the films as depicted in Figure 4d. This intriguing finding is counterintuitive and unreported so far, for example, for porous Si^[^ ^53^ ^]^ one finds decreasing *c*_L_(ϕ) = *c*_L_(0)(1 − ϕ^*x*^) with 0.6 < *x* < 1.3 depending on crystallographic orientation. In view of the decreasing overall density with porosity (Figure 4d) this unexpected *c*_L_(ϕ) trend reflects strong chain stretching/coupling. On the account of the isotropic sound propagation (Figure 4c) chain stretching should be direction independent, in agreement with simulations showing that chains extend over several pores.

The increase of *c*_L_ with porosity suggests phonon propagation through the stretched PS chains essentially ignoring the air in the nanopores.^[^ ^54^ ^]^ Thus the PS melt density was used to calculate the moduli. These shear *G*_PS_ and Young's *E*_PS_ moduli for the PS frame increase with porosity as shown, respectively in Figure 4e,f revealing an unprecedented reinforcement due to chain expansion. The bulk modulus *K*_PS_ is virtually insensitive to the porosity (Figure 4e). Due to the selective phonon propagation, BLS allows to determine the frame elasticity otherwise inaccessible for example, by macroscopic tensile testing. The effective medium moduli, *E*_eff_, *G*_eff_, and *K*_eff_ estimated from corresponding frame moduli times (1 − ϕ) expectedly drops with porosity (Figure 4e,g). Poisson's ratio is determined solely from the sound velocities ratio *c*_L_/*c*_T_. It is found to decrease with porosity due to the stronger increase of *c*_T_(ϕ) than *c*_L_(ϕ) (Figure 4f) and reaches values typical for polymer foams.^[^ ^55^ ^]^ Poisson's ratio ν measures how films shrink normal to an in‐plane applied stretching. A decrease below the PS value implies reduced shrinking upon stretching.

While experiments so far were conducted at room temperature, deep in the glassy state of the PS films, BLS also can be used to study their thermomechanical behavior harnessing the temperature dependence of the LA phonon at *q*_∥_ = 0.0167 nm^−1^ (see Figure S12, Supporting Information). In addition, the refractive index *n*(*T*) obtained from the LA at back scattering *q*_BS_ = 4π*n*/λ can be utilized to estimate ϕ(*T*). Figure 4g shows *c*_L_(*T*) of PS films with three different porosities and bulk PS (ϕ = 0) for comparison. For the latter, *c*_L_ shows the well known linear decrease with temperature,^[^ ^56^ ^]^ with two different rates identifying *T*_g, bulk_ ≈ 380 K. *c*_L_ of the porous films (ϕ > 0) monotonically decreases with increasing temperature and gradually approaches the PS bulk *c*_L_ value at *T* ≈ *T*_g, bulk_. While the lowest porosity film resembles the temperature dependence of bulk PS with change of the slope d*c*_L_/dT at *T*_g, bulk_, films of higher porosity show a stronger gradual softening at temperatures *T* < *T*_g, bulk_, shifting to lower temperatures with increasing porosity. This stronger softening, however, even for the P‐40 sample sets in only for *T* above about 330 K. At *T* > *T*_g, bulk_, *c*_L_ in the three originally porous films is that of bulk PS and displays the same linear temperature‐dependence. To support this distinct *c*_L_(ϕ, *T*) behavior of the porous films, the temperature‐dependent porosity of three films is shown in the inset of in Figure 4g. With increasing temperature the porosity is quite robust up to an intermediate porosity‐dependent softening temperature *T*_s_^[^ ^57^ ^]^ above which it strongly decreases and practically reaches zero at about *T*_g, bulk_. Importantly both sound velocity and porosity slowly change with increasing temperature to eventually merge results from bulk polystyrene around the bulk glass transition temperature of about 380 K.

## 4 Conclusion

We have shown, first by simulation and then by experiment, that stable well controlled nanoporous films (or nanoporous foams) can be made just by mechanical deformation of highly entangled polymer films and a subsequent quench and stabilization of this non‐equilibrium material below *T*_g_. Simulations reveal that these films rely on long chains, which extend over several bridges between pores. Keeping that in mind, polydispersity does not destroy the effect. Large polydispersity, however, changes the terminal relaxation times of the melts, which could introduce some additional lower limits for the deformation strain rates. The comparison with X‐ray scattering, based on a length scale normalization of the scattering vector by the respective reptation tube diameters, shows a semi quantitative agreement between simulation and experiment as well as with SEM, SFM, and BLS. Mechanical properties as determined by BLS display remarkable features, namely an increase of the sound velocity, changed elastic constants and a reduced Poisson ratio in comparison to an equilibrium melt. BLS experiments also demonstrate that the glassy structure is needed to conserve the porous medium, since the measured sound velocity and so on obtain standard bulk values upon heating of the porous films toward *T*_g_. The good consistency between simulation of a simple bead spring model and experiment with PS based on a unified length scale suggests that porous films with larger/smaller pores can be fabricated in a controlled way by varying the expansion protocol and by using other polymers with different *d*_T_. The only strict requirement for a broader application of this approach to other amorphous polymers is the need for chains significantly longer than *N*_e_ and an expansion process above *T*_g_ with a subsequent quench below *T*_g_ to freeze the porous structure.

In principle this whole procedure can also be applied to crosslinked melts, as long as the typical distance between crosslinks along the chain contour is significantly larger than *N*_e_, *M*_e_, respectively. A high crosslinking density will make the possibility to form nanoscopic pores as the ones shown here without any chain scission very unlikely, if not impossible. Then the same arguments as above apply, with the difference that the upper limit for time scales, that is, slowness of expansion, is arbitrary large for an ideally crosslinked system.

## Conflict of Interest

The authors declare no conflict of interest.

## Author Contributions

H.P.H. performed molecular dynamics simulations, analyzed data, and wrote the paper; M.S. made all experimental systems, BET experiments, analyzed data, and wrote the paper; K.K. initiated and designed the project, analyzed data, and wrote the paper; Y.C. and G.F. performed Brillouin light scattering (BLS) and G.F. wrote the paper; H.T.‐A. provided lab equipment to make samples and helped in making samples; M.M. performed SAXS measurements and SAXS data analysis; I.L. performed SEM and R.B. performed SFM experiments.

## Supporting information

Supporting Information

## Data Availability

The data that support the findings of this study are available from the corresponding author upon reasonable request.
