# Supplementary material for: Free Standing Dry and Stable Nanoporous Polymer Films Made through Mechanical Deformation
Source: Adv Sci (Weinh). 2023 Apr 25;10(18):2207472. doi: 10.1002/advs.202207472 (PMC10288257; doi:10.1002/advs.202207472)
Supplement: Supplementary file 1 — Supporting Information [file ADVS-10-2207472-s001.pdf]

ADVANCED SCIENCE
Open Access

Supporting Information

for *Adv. Sci.*, DOI 10.1002/advs.202207472

Free Standing Dry and Stable Nanoporous Polymer Films Made through Mechanical Deformation

*Hsiao-Ping Hsu, Manjesh K. Singh, Yu Cang, Héloïse Thérien-Aubin, Markus Mezger, Rüdiger Berger, Ingo Lieberwirth, George Fytas and Kurt Kremer**

Supporting Information: Free standing dry and stable nanoporous polymer films made through mechanical deformation

Hsiao-Ping Hsu[1], Manjesh K. Singh[1,2], Yu Cang[1,3], Héloïse Thérien-Aubin[1,4], Markus Mezger[1,5], Rüdiger Berger[1], Ingo Lieberwirth[1], George Fytas[1] and Kurt Kremer[1*]

[1]Max-Planck-Institut für Polymerforschung,Ackermannweg 10, Mainz, 55128, Germany.
[2]IIT Kanpur,Kanpur: 208016,Uttar Pradesh,India.
[3]School of Aerospace Engineering and Applied Mechanics, Tongji University, Zhangwu Road 100, Shanghai, 200092, China.
[4]Chemistry Department, Memorial University of Newfoundland, St. John's, NL, Canada.
[5]University of Vienna, Faculty of Physics, Boltzmanngasse 5, 1090 Wien, Austria.

*Corresponding author(s). E-mail(s):
kremer@mpip-mainz.mpg.de;
Contributing authors: hsu@mpip-mainz.mpg.de;
singhm@mpip-mainz.mpg.de, manjesh@iitk.ac.in;
yucang@tongji.edu.cn, yucang@tongji.edu.cn;
therien@mpip-mainz.mpg.de, htherienaubin@mun.ca;
mezger@mpip-mainz.mpg.de,markus.mezger@univie.ac.at;
berger@mpip-mainz.mpg.de; lieberw@mpip-mainz.mpg.de;
fytas@mpip-mainz.mpg.de;

Contents

1 Simulation: Modelling Details **2**
1.1 Model 2
1.2 Biaxial expansion at $T = 1\epsilon/k_B$ 3
1.3 Cooling 4
1.4 Porosity and pore size distribution 5
1.5 Morphology of monodisperse and polydisperse porous films upon fast expansion 5

2 Experimental Procedures **6**
2.1 Systems 6
2.2 BET Experiments 8
2.3 Scanning Electron Microscopy (SEM) 9
2.4 Scanning Force Microscopy (SFM) 10
2.5 Small Angle X-ray Scattering (SAXS) 11
2.6 Brillouin light scattering (BLS) 12

1 Simulation: Modelling Details

1.1 Model

For our simulations, $n_c = 1000$ polymer chains of length $N = 2000$ monomers in free-standing polymer films are modelled by a recently developed modified coarse-grained model [1, 2]. For that attractive and bond bending contributions are added, while the original standard interactions [3, 4] are kept. I.e. any pair of bonded and non-bonded monomers located at a distance r interacts via a shifted, purely repulsive Lennard-Jones (LJ) potential $U_{\text{LJ}}(r)$ with the cutoff in the minimum at $r_{\text{cut}} = 2^{1/6}\sigma$. Here $\sigma = 1$ is the LJ unit of length and the size of monomers, ϵ is the LJ unit of energy, also providing a natural time definition $\tau = \sigma\sqrt{m/\epsilon}$, where $m = 1$ is the mass of monomers. Any pair of bonded monomers in addition interacts via the finitely extensible nonlinear elastic (FENE) binding potential [5] $U_{\text{FENE}}(r)$ with the force constant $k = 30\epsilon/\sigma^2$ and the upper bound of bond length $R_0 = 1.5\sigma$. Such a melt typically exerts a pressure weakly below $P \approx 5\epsilon/\sigma^3$ at standard density $\rho = 0.85\sigma^3$. By adding a bond bending potential the entanglement length N_e is adjusted. Theoretical predictions of dynamics of chains in melts related to N_e, or molecular weight M_e given by the tube/reptation model [6] have been verified in the literature [3, 4, 7–18]. Usually a bond bending potential of the form $U^{\text{(old)}}_{\text{BEND}} = k_\theta(1 - \cos\theta)$ between subsequent bonds [19–22] is introduced. With $k_\theta = 1.5\epsilon$ one models

weakly semiflexible chains at temperature $T = 1\epsilon/k_B$ with a Kuhn length of $\ell_k \approx 2.66\sigma$ and $N_e = 28$, as widely used in the literature [17, 22–24]. Thus, for $N = 2000$ we observe a squared end-to-end distance $\langle R^2\rangle \approx 5454\sigma^2$ and a squared radius of gyration $\langle R_g^2\rangle \approx 909\sigma^2$ [17].

The constraints on the motion of monomers are characterized by the reptation tube of diameter d_T, which is determined by the effective coil extension of a chain of length N_e. In Ref. [17] we derive d_T from the value of the monomer mean square displacement at the crossover from Rouse to reptation dynamics, namely the Rouse relaxation time $\tau(N_e)$ of a chain of length N_e, which is at a displacement of $d_T^2 = 2 < R_g^2(N_e) >$. Ref [25] instead defines d_T as $d_T^2 =< R^2(N_e) >$. With the general relation $R^2 = 6R_g^2$ for Gaussian chains, which is well fulfilled for the present systems, there is a factor of $\sqrt{3}$ between the respective values quoted [18]. This does not affect any conclusion, but should be kept in mind for quantitative comparisons.

For studying temperature dependent properties this choice has the disadvantage that chains are fully stretched in the ground state. In contrast, polymer conformations only weakly change as a function of temperature in amorphous systems. Thus we replace the old bond bending potential by $U_{\mathrm{BEND}}(\theta) = -a_\theta \sin^2(b_\theta\theta)$ for $0 < \theta < \theta_c = \pi/b_\theta$ with $a_\theta = 4.5\epsilon$ and $b_\theta = 1.5$ [1, 2]. At $T = 1\epsilon/k_B$ we obtain conformations indistinguishable from the ones with the conventional bending potential, allowing a seamless switch of models. For free surfaces, the internal pressure has to be zero and a short-range attractive potential between non-bonded monomers $U_{\mathrm{ATT}}(r) = \alpha\cos(\pi\,(r/r_{\mathrm{cut}})^2) - \alpha$ for $r_{\mathrm{cut}} \le r < r_c^a$ where $U_{\mathrm{ATT}}(r) = 0$ at the upper cut-off $r = r_c^a$ is introduced [1, 2, 26]. At $r = r_{\mathrm{cut}}$ and at $r_c^a = \sqrt{2}r_{\mathrm{cut}} \approx 1.5874\sigma$ forces vanish, avoiding any algorithmic instabilities. Setting $\alpha = 0.5145\epsilon$, a polymer melt of long chains with free surfaces at $P = 0\epsilon/\sigma^3$ equilibrates at the standard melt density of $\rho = 0.85\sigma^{-3}$. For $n_c = 1000$ chains of $N = 2000$ monomers this gives for polymer films of thickness $h \approx 130\sigma$ a lateral extension $L_{x,y} \approx 134\sigma$, as studied here.

Free-standing films have been equilibrated in an efficient way based on a hierarchical backmapping strategy [27]. First, films are confined between two soft repulsive walls and equilibrated in their coarse-grained representation where polymer chains are treated as strings of Gaussian blobs. Then we apply a (one-step) backmapping to reinsert the microscopic details of the underlying bead-spring model. For taking care of equilibration of the local bead packing, only short MD simulations about $6\tau_e$ independent of chain length N are performed. Finally, after switching on $U_{\mathrm{ATT}}(r)$ mentioned above, we can prevent system instability and remove the walls at $P = 0\epsilon/\sigma^3$.

1.2 Biaxial expansion at $T = 1\epsilon/k_B$

Starting from the fully equilibrated free-standing films we apply a simple "biaxial expansion" deformation to stretch the film at a slow strain rate $\dot{\varepsilon}\tau_{R,N} = 77$ into the two lateral dimensions with periodic boundary conditions up to a strain of 4×4. Technically the films are first instantaneously

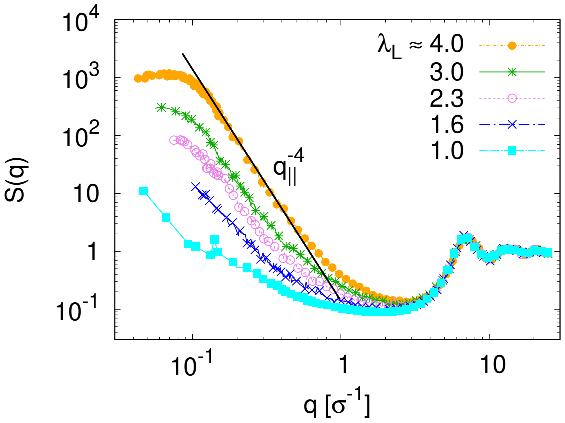

Figure S1 In-expansion-plane structure factor: Structure factor $S(q)$ as a function of q for simulated expanded film at several selected expansion ratios of λ_L at $T = 1.0\epsilon/k_B$, as indicated.

stretched by a factor of 1.02 along the x, and then along the y-direction. After each instantaneous stretch, the lateral dimensions are fixed and system can relax for $(0.02\tau_{R,N}/77)\tau \approx 1.3\tau_e$ giving an effective strain rate of $\dot{\varepsilon} = 77/\tau_{R,N} = 0.015/\tau_e$. The strain rate is slow enough, so that in between the stretching steps the pressure tensor normal to the interfaces, P_{zz}, always relaxes to zero within the error bars. Iterating this until a strain of 4×4 is reached, results in films of thickness $h(\approx 10.02\sigma)$ in an off-equilibrium state at $P_{zz} \approx 0\epsilon/\sigma^3$. These films actually are thinner than the equilibrium chain extension in bulk, where the radius of gyration $R_g = \langle R_g^2 \rangle_0^{1/2} \approx 30.15\sigma$ [17]. $\langle \cdots \rangle_0$ stands for the average over all chains in bulk. Tests with periodic boundary conditions in all three Cartesian directions result in the qualitatively same result, when thin slices of similar thickness are analyzed. Polymer conformations in the expanded films globally follow the anticipated affine deformation as shown in the previous studies [28–30]. Molecular dynamics (MD) simulations with Langevin thermostat were carried out using the ESPResSO++ package [31, 32]. The MD time step is set to $\Delta t = 0.01\tau$, and the thermostat friction coefficient $\Gamma = 0.5\tau^{-1}$. Figure S1 shows the scattering function $S(q)$ from simulation data for the unperturbed ($\lambda_L = 1$), and four selected stretching ratios of $\lambda_L \approx 1.6$, 2.3, 3.0 and 4.0, respectively. The q^{-4} Porod scattering power law, indicating sharp well defined polymer void interfaceskk of the pores, is best expressed for the case of $\lambda_L = 4$.

1.3 Cooling

To study the interplay of topological constraints and void formation after film expansion the films are quenched at a cooling rate $\Gamma = \Delta T/\Delta t = 8.3 \times 10^{-7}\epsilon/(k_B\tau)$ to $T = 0.5\epsilon/k_B$, well below the glass transition temperature $T_g \approx 0.67\epsilon/k_B$ [1, 2, 33]. Technically the temperature is reduced in small steps

of $\Delta T = 0.025\epsilon/k_B$ from $T = 1.0\epsilon/k_B$ to $T = 0.2\epsilon/k_B$ with a relaxation time between each step of $\Delta t = 30000\tau \approx 13\tau_e$. Again the NVT ensemble with Langevin thermostat of the ESPResSo++ [31, 32] package is used. Throughout this procedure h can freely adjust and P_{zz} of the thin porous films remains at $P_{zz} = 0.0\epsilon\sigma^{-3}$.

1.4 Porosity and pore size distribution

To estimate the porosity ϕ and pore size distribution $P(d_p)$ of simulated films, we adopt the definition by Gubbins et al. [34–37] where ϕ and $P(d_p)$ depend on the accessible volume of a spherical test particle. Assuming a hard sphere test particle of a monomer size (1σ diameter), the void space V_{void} is determined by inserting the test particle in the total effective volume $V_{\text{film}} = hL_xL_y$ of film. Here $h = z_G^{(\text{upper})} - z_G^{(\text{lower})}$ is the film thickness, i.e. the distance between the upper and lower bounds of the free-standing film according to the concept of Gibbs dividing surface [38–40]. The porosity $\phi = \frac{V_{\text{void}}}{V_{\text{film}}} \times 100\%$, is the percentage of void volume V_{void} in total volume V_{film} of the film. We practically estimate ϕ of our systems by simply performing a Monte Carlo (MC) integration of V_{void}. The radius R of any pore, assuming a spherical shape in the void space, $d_p = 2R$, is determined by first randomly selecting a point $\vec{r}_p$ in the void space, and then find the largest radius R of sphere located at $\vec{r}_c$ containing $\vec{r}_p$ in the void space satisfying the conditions: [37, 41]

$$R(\vec{r}_c) = \text{Min.}(d_{0c}, d_{1c}, \ldots, d_{N_{\text{tot}}c}) - \frac{1}{2}\sigma \quad \text{and} \quad \mid \vec{r}_c - \vec{r}_p \mid \leq R(\vec{r}_c)\,, \tag{1}$$

where $d_{ic} =\mid \vec{r}_i - \vec{r}_c \mid$ is the distance between monomer i and the center of sphere, $\vec{r}_c$. The distribution $P(d_p) = -\frac{dH(d_p)}{dd_p}$ is thus given by the negative derivative of the cumulative histogram $H(d_p)$ that counts the probability of finding a point in the void space with a pore size equal and smaller than d_p.

1.5 Morphology of monodisperse and polydisperse porous films upon fast expansion

The stabilization of the nanoporous structure requires chains, which are long enough to extend over several bridges between pores. While this is essential, it does not require monodisprese films. To demonstrate that our method can also be applied for the generation of stable polydisperse porous films, we follow the same strategies described in Sec. 1.2, and Sec. 1.3, but starting from a fully equilibrated free-standing film of a polydisperse polymer melt. For this test we use $n = 1000$ chains of $N_1 = 1900$ monomers, and $n = 1000$ chains of $N_2 = 100$ monomers, giving $M_N = 1000$ and $M_W/M_N = 1.8$). For this quick test the polydisperse polymer film is expanded at a faster expansion rate comparing to the strain rate mentioned in Sec. 1.2, $\dot{\varepsilon}\tau_{R,N_1} \approx 29000$, until a strain of 4×4 is reached. Though faster, this strain rate still allows relaxataion during stertching on scales of the order of the tube diameter. For comparison, we also

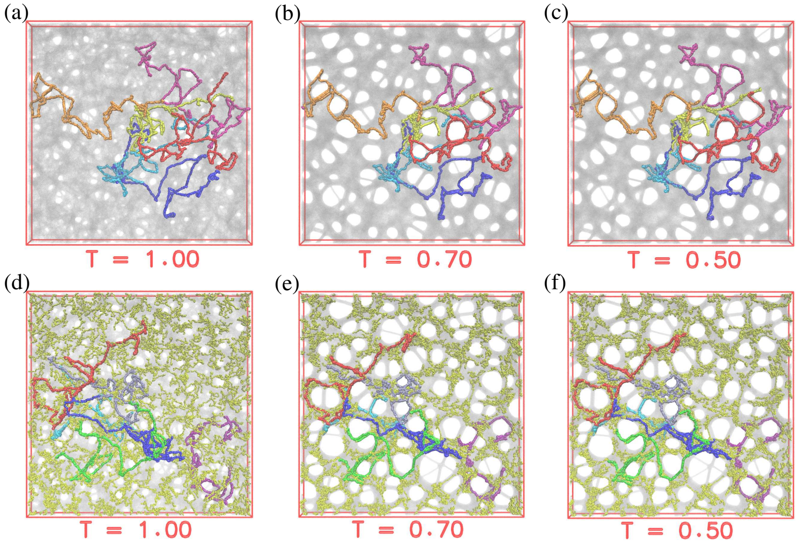

Figure S2 Porous film morphology. Simulated expanded films of monodisperse (a)-(c) and polydisperse polymers (d)-(f) at $T[\epsilon/k_B] = 1.0$ (a)(d), 0.7 (b)(e), and 0.5 (c)(f). Six randomly selected chains of $N = 2000$, and $N_1 = 1900$ are marked in (a)-(c), and (d)-(f), respectively. In (d)-(f), all 1000 chains of $N_2 = 100$ are also marked in yellow color. At $T = 0.5\epsilon/k_B < T_g$, the film thickness h, porosity ϕ and average pore size D_{pore} is about $h \approx 9.8\sigma$, $\phi \approx 29\%$, $D_{\text{pore}} \approx 22\sigma$ in (c), and $h \approx 10.4\sigma$, $\phi \approx 33\%$, $D_{\text{pore}} \approx 25\sigma$ in (f).

stretch a monodisperse polymer film (1000 chains of $N = 2000$ monomers) at a compatible fast strain rate $\dot{\varepsilon}\tau_{R,N} \approx 32000$. Figure S2 shows the morphological changes of expanded monodisperse and polydisperse polymer films upon cooling. Both display a very similar structure, with slightly larger pores for the polydisperse system. Here, however, the observed clustering of the short chains point to some strain induced segregation between the strained long chains and the short chains, which can relax almost completely. Obviously this does not affect the stability of the porous film.

2 Experimental Procedures

2.1 Systems

For all experiments we have used monodisperse atactic polystyrene (PS) of $M_n = 1\times10^6$ Da (polydispersity PDI= $M_w/M_N = 1.04$). The PS was obtained from Sigma Aldrich (Germany). Figure S3 shows the weight distribution of the PS as obtained from GPC experiments.

PS films were prepared by pressing 250 mg of PS under 20 kN load and temperature 433 K well above [42] $T_g = 380$ K, for an hour. This yielded well equilibrated transparent films of about 100 μm thickness. To deform the film with a biaxial stress, the PS film was immobilized by the edges over circular aperture of 1.75 cm of diameter and deformed by applying a load using the negative pressure generated by a controlled vacuum of 50 mbar. This was

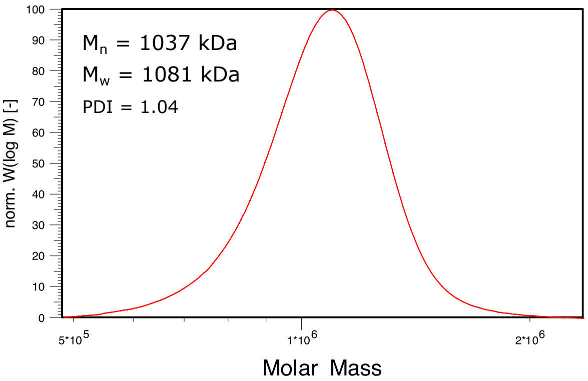

Figure S3 Molecular weight distribution of PS used in the current work.

achieved by attaching the film to one end of a vacuum flange using a cyanoacrylate glue. The flange was put in an oven and maintained at a temperature of 473 K. After 2 min., the vacuum pump was switched on and a vacuum of approximately 50 mbar was maintained for different times of duration. Films were deformed radially and form a hat-like structure, (Figure 1b main text). Depending on the duration of vacuum pressure applied, different degrees of stretching were achieved in PS films as given in Table S1. The time scales involved are significantly larger than simulation times, however still very short compared to the overall chain relaxation time. To preserve the morphology of the stretched films, they are quenched far below T_g by dipping them into liquid nitrogen. After that films were kept at room temperature without any special conservation precautions.

Table S1 Sample types after different degree of stretching.

Sample	Thickness before stretching (μm)	Duration of Stretching (mins)	Thickness after stretching (μm)	Diameter (mm)	Mass (mg)	Density (g/cm^3)
P-0	91.0	0	91.0	5.0	1.759	0.985
P-10	110.0	15	106.0	5.0	1.958	0.941
P-26	80.0	21	40.0	5.0	0.655	0.819
P-40	100.0	30	12.0	5.0	0.174	0.732

Table S1 shows the details of 4 different films (stretched and unstretched). From the thickness and the weight of small cut out 5 mm diameter disks at the hat centers the density is found to be reduced to about $\rho = 0.732\,\mathrm{g/cm^3}$ compared to the standard melt value of $1.05\,\mathrm{g/cm^3}$. For the unstretched film, the measured $0.985\,\mathrm{g/cm^3}$ indicated that the pristine films were not completely homogeneous (Table S1). We note in passing that if there were any prior fault/holes present in the unstretched film, then on application of vacuum, the film would have burst in few seconds. Most experiments were performed

on P-40 systems, which most closely correspond to the simulation systems.

2.2 BET Experiments

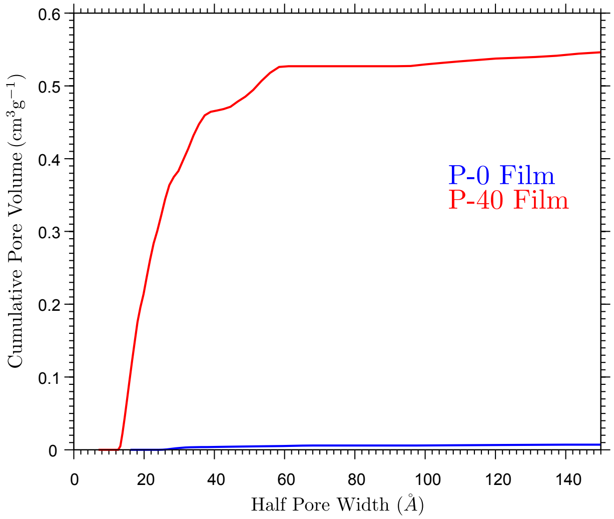

Figure S4 BET Experiments: Comparison of degree of porosity in P-0 and P-40 films.

To confirm and complement the porosity analysis of the expanded samples, we have performed gas-adsorption (BET) experiments [43], on unstretched P-0 film in Table S1) and highly-stretched (P-40 film in Table S1) PS films. The adsorption experiments were performed for N_2 gasses at 77 K. The results in terms of comparison of cumulative-pore-volume for the P-0 and P-40 films are presented in Figure S4. The pore volume in the sample P-40 is $0.552\,\mathrm{cm}^3\mathrm{g}^{-1}$ whereas it is about a factor of 70 lower, namely about $0.008\,\mathrm{cm}^3\mathrm{g}^{-1}$ for the sample P-0 film. Considering a approximate PS melt density of $1\mathrm{g}/\mathrm{cm}^3$ these results are in good agreement with the porosity estimated from BLS experiments for P-40 films in Table S1. The BET technique was developed to estimate the total surface area of solid powders. It does not directly measures pore sizes or pore size distributions. Typically, one obtains an estimate of the total pore volume from the volume of nitrogen taken up at a pressure of 1 atm and a temprature of 77 K. Similarly, the BET surface area measurements provide estimate of total surface area of the pores. Assuming cylindrical pores, the total pore volume $V(\mathrm{BET}) = \pi d^2 l/4$ and BET surface area $S(\mathrm{BET}) = \pi dl$. Here d is the mean pore diameter and l is the total length of the pores. Using the above two equations, diameter d of pores is calculated by eliminating l.

2.3 Scanning Electron Microscopy (SEM)

For SEM imaging, an approximately $5 \times 5\,\mathrm{mm}^2$ piece was cut out from the central hat area. This piece was immersed in liquid nitrogen for a few seconds and then brittle fractured into two pieces. The fracture surface was then mounted in a 90° cross-section SEM holder and examined in a LEO Gemini 1530 SEM. The picture shown from SEM experiments thus shows a plane perpendicular to the expansion plane, while the simulation view is a top view down onto the expansion plane. The sample surface was not sputtered with a conductive layer to avoid covering any small pores and to image the fracture surface as pristine as possible. Typical imaging conditions are a working distance of 1.3 mm at an electron landing energy of 250 eV using the in-lens secondary electron detector. Images explaining the sample preparation are shown in Figure S5.

To prove that the observed nanoporous structure is not an artefact of the sample preparation we also show in Figure S6 an SEM image of a surface of an unstretched PS film, prepared in exactly the same way as the stretched ones. As can be seen, we get a homogeneous smooth surface and there are almost no holes at all. Only in apparently strongly bent regions one can observe some pores, which are much smaller than the ones in the strained samples.

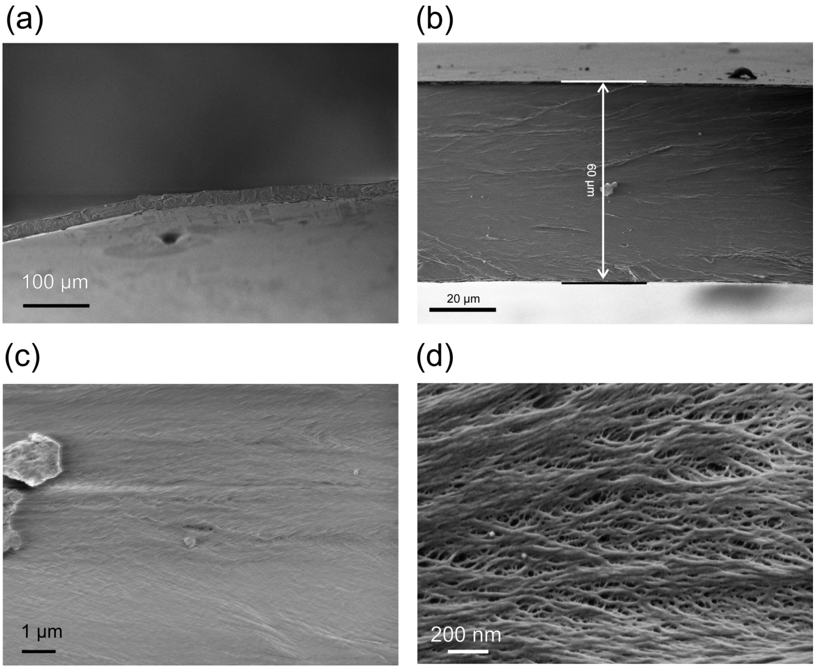

Figure S5 SEM micrographs of the fracture surface of the biaxial expanded PS film. (a) and (b) show the fracture surface of the film in top view at low magnification. Here, the film thickness is around 60 μm, which indicates that the area measured was not perfectly extracted from the top of the “hat”. However, even at this slightly off-axis position the porous structure of the expanded film clearly formed over larger areas (c), as shown by the zoom-in in (d).

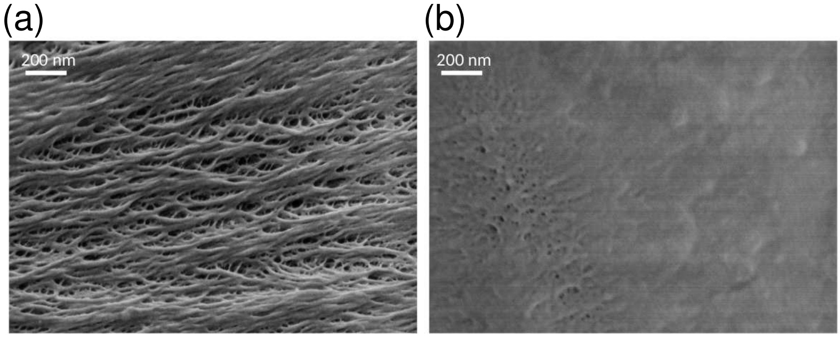

Figure S6 Characteristic SEM micrographs of fracture surfaces of (a) and expanded and (b) an unstretched film, as they are later on used for biaxial expansion. Both figures are at the same magnification.

2.4 Scanning Force Microscopy (SFM)

SFM is a well established tool to analyze surface structures in detail. For SFM we used fracture surfaces as also used for SEM, which were mounted in a 90° cross-section SEM holder. We have performed SFM experiments (Dimension ICON, Bruker) in tapping mode on surfaces, which have been cut by a diamond knife (Microtome UC6 from Leica, knife from Diatome). More details are given in Figure S7.

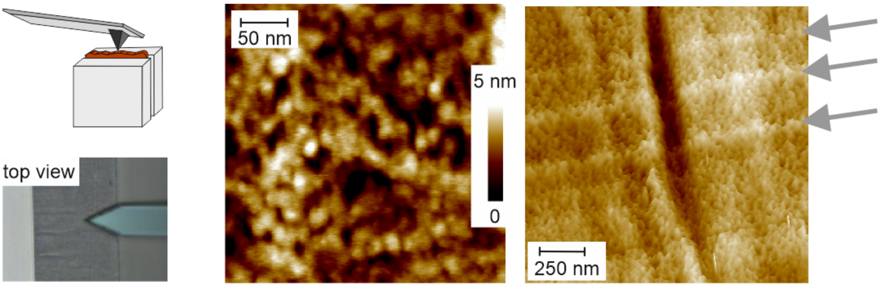

Figure S7 We have performed SFM experiments (Dimension ICON, Bruker) in tapping mode on surfaces, which have been cut by a diamond knife (Microtome UC6 from Leica, knife from Diatome). The film was left in the holder of the microtome and the film protrudes by approx. 100 μm from the holder surface (top left image). The top view image shows the SFM cantilever (OPUS 160 AC NA, Nano and More, Germany) on the cut section of the film. We performed all measurements close to the center of the film. The high resolution SFM topography exhibits a granular structure with feature diameters down to 10 nm. At lower magnification stripes are visible which are caused by unevenness of the blade of the cutting knife (arrows in right SFM topography image).

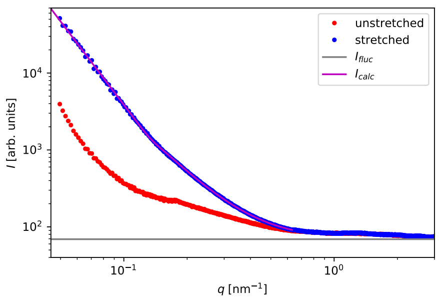

Figure S8 Radial averaged SAXS data for the stretched (blue) and unstretched (red) PS samples. The gray line represents the scattering contribution from soft matter density fluctuations. The magenta curve is the fit of two individual power laws with a smooth transition at $q = 0.64\,\mathrm{nm}^{-1}$.

2.5 Small Angle X-ray Scattering (SAXS)

SAXS data were obtained on a home-built device using Cu Kα radiation (wavelength $\lambda = 1.54\mathring{A}$) from a rotating anode source (Rigaku MicroMax 007 X-ray generator) with curved multilayer optics (Osmic Confocal Max-Flux) and three 4-jaw slit sets ($1 \times 1\,\mathrm{mm}^2$) providing 150 cm collimation length. Stacks of polymer foils were measured in normal incidence transmission geometry with 80% and 70% direct beam transmission for the stretched and non-stretched samples, respectively. In this geometry, the momentum transfer q at the scattering angles 2θ investigated lie almost parallel to the sample surface. The scattered intensity was recorded on a 2D detector (Mar345 image plate) at 1.45 m sample detector distance calibrated with silver behenate as reference. Three independent SAXS measurements with 900 s exposure time each were averaged and corrected by dark images. Artifacts, originating from high energy radiation, were removed by Laplace filtering. 2D data sets were converted to $I(q)$ vs momentum transfer $q = 4\pi/\lambda \sin\theta$ by radial averaging. SAXS data from the stretched and unstretched samples were rescaled to overlap in the high $q > 0.64\,\mathrm{nm}^{-1}$ region.

To account for the soft matter density fluctuation background, a constant contribution I_{fluc} was subtracted from the scattering data (Figure S8). This subtraction maximizes the extent of the linear regime in the Kratky plot $I(q)q^2$ vs q (Figure S9). At the minimum of $I(q)q^2$ (q= $0.64\,\mathrm{nm}^{-1}$), I_{fluc} corresponds to 77% of the total scattered intensity from the stretched sample. For higher q, were $I(q)q^2$ starts increasing towards the amorphous halo, we find a good agreement between the signals from stretched and unstretched samples. Deviations between the experimental and simulated patterns in the q-range around the amorphous halo are expected since one structureless coarse grained simulation bead corresponds to roughly 6 PS monomers.

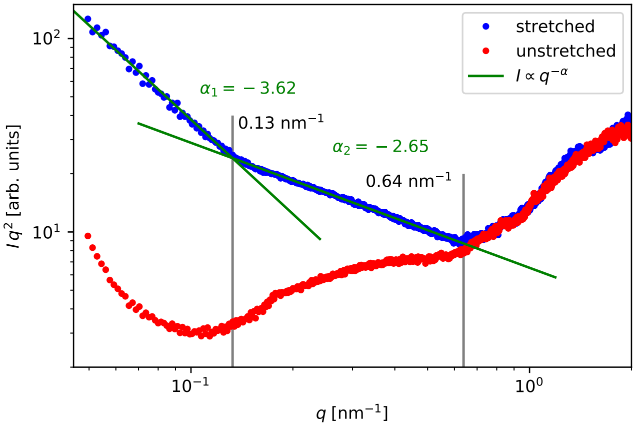

Figure S9 Kratky plot of the fluctuation background corrected and rescaled SAXS data from the stretched (blue) and unstretched (red) PS samples. The green lines represent power-laws $I \propto q^{\alpha}$ with exponents $\alpha = \alpha_1 = -3.62$ and $\alpha = \alpha_2 = -2.65$, respectively. Grey vertical lines indicate the two transitions between the low $q = 0.13\,\mathrm{nm}^{-1}$ and high $q = 0.64\,\mathrm{nm}^{-1}$ of the power-law regimes.

2.6 Brillouin light scattering (BLS)

The frequency of scattered light from propagating thermal phonons depends on their wave vector $\mathbf{q}$ and the sound velocity, c, in the medium and is Doppler shifted by $\pm f$ around the frequency of the incident monochromatic light of wavelength $\lambda(= 532\,\mathrm{nm})$. The probed $\mathbf{q} = \mathbf{k}_s - \mathbf{k}_i$ is defined by the scattered ($\mathbf{k}_s$) and incident ($\mathbf{k}_i$) light wavevectors, forming a preselected scattering angle, $10^o < \theta < 150^o$ by means of goniometer rotation of the incident laser beam around the optical axis (TFPI axis and incident laser focus) of the sample cell [44]. The frequency shift $f = \pm cq/(2\pi)$ is resolved by a high-resolution tandem Fabry-Pérot interferometer (TFPI of JRS Instruments). $q = (4\pi n/\lambda)/\sin(\theta/2)$, in general depends on the refractive index (n), scattering angle (θ), and the polarization of the probed phonons, which is selected by polarization combination of incident and scattered light. Longitudinal (LA) and transverse (TA) acoustic phonons are probed for parallel (VV) and crossed (VH) polarized light configuration, respectively. The first letter (V) denotes vertically polarized incident light, whereas the second letter V(H) is vertically (horizontally) polarized scattered light with respect the scattering plane, $(\mathbf{k}_i, \mathbf{k}_s)$. Depending on the polarization of the probed phonons, the measured sound velocity from the BLS spectra can be either the longitudinal, c_L or in the case of solids transverse c_T.

The n-dependence of q can be bypassed in the specific transmission geometry for which the incidence laser angle, $\alpha = \theta/2$, and $\mathbf{q} = \mathbf{k}_s - \mathbf{k}_i$ directs

in the film plane with magnitude $q_{\|} = 4\pi \sin\alpha/\lambda$ (Figure 3a). In the reflection geometry (Figure S10) $\mathbf{q}$ directs normal to the film plane with $q_{\perp} = 4\pi\sqrt{(n^2 - \sin\alpha^2/\lambda)}$ depending on n normal to the plane.

For temperature scan measurements, the sample temperature is monitored with a platinum resistance temperature detector and controlled with a homemade temperature controller. The sample was allowed to isothermally equilibrate for 20 min before recording spectra [45].

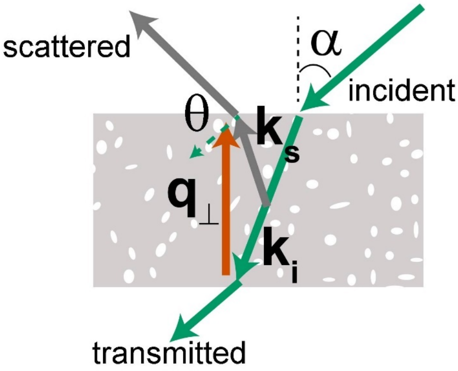

Figure S10 Schematic of reflection geometry. The wavevector $\mathbf{q}_{\perp} = \mathbf{k}_s - \mathbf{k}_i$ is normal to the film plane when incident angle $\alpha = 90^o - \theta/2$, where $\mathbf{k}_s$ and $\mathbf{k}_i$ are scattered and incident lights, respectively, and θ is the scattering angle. The magnitude of the phonon wavevector, $\mathbf{q}_{\perp} = 4\pi\sqrt{n^2 - \sin\alpha^2}/\lambda$ depends on the medium refractive index n.

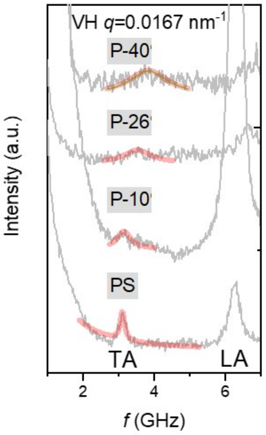

Figure S11 Depolarized VH BLS spectra recorded at $q = 0.0167\,\mathrm{nm}^{-1}$ for bulk and porous PS films. The spectra (grey lines) are represented by single Lorentzians (red lines). The low- and high- frequency peaks are assigned to transverse (TA) and longitudinal (LA) acoustic modes, respectively.

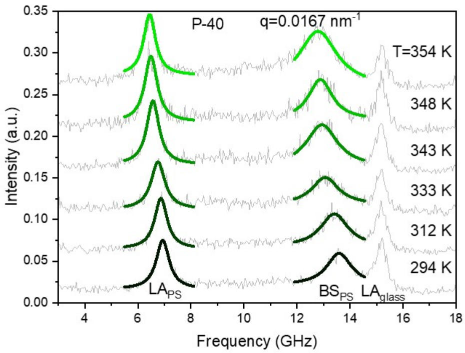

Figure S12 Polarized VV BLS spectra for porous PS film (P-40) with 40% porosity at recorded $q = 0.0167\,\text{nm}^{-1}$ at different temperatures. The observed three peaks are assigned to longitudinal acoustic modes of PS frame (LA_{PS}) and glass (LA_{glass}) respectively, and backscattering mode of PS frame (BS_{PS}). The longitudinal acoustic (LA_{PS}) and back scattering (BS_{PS}) of the P-40 film are represented by Lorentzians in green. The peak at the highest frequency is the LA_{glass} phonon in the glass substrate.

Nanopore space correlation

The dispersion of the mode 2 frequency is described by $f_{\text{mode}\,2} = (c/2\pi)(q+G)$, where $G = 2\pi/d$ and d denotes an effective nanoporous periodicity. The anticipated $d \sim \phi^{-1/3}$ is probable giving the large error.

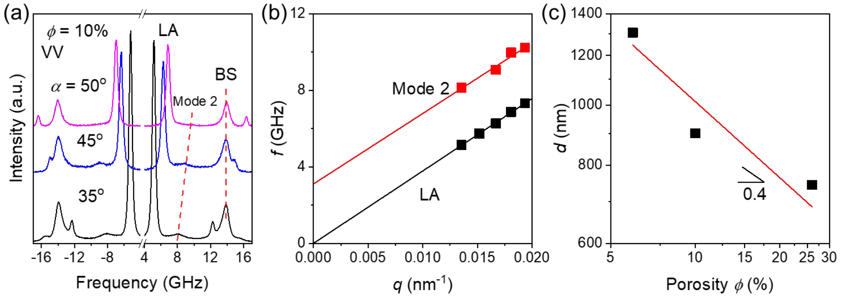

Figure S13 (a) Polarized BLS spectra of porous PS film (P-10) with porosity 10% at three different q's in the plane of the film. From the high to the low frequency, BS longitudinal phonon with robust frequency, an unexpected weak peak (mode 2) and the dominant longitudinal (LA) phonon in the film. (b) The expected linear acoustic dispersion of the LA phonon (black square) and the linear dependence of mode 2 with finite intercept. (c) The characteristic spacing d decreases porosity approximately according to $d \sim \phi^{-0.4\pm0.1}$. The dashed lines are a guide to the eye and solid lines represent the linear fits.

References

[1] Hsu, H.-P., Kremer, K.: A coarse-grained polymer model for studying the glass transition. J. Chem. Phys. **150**, 091101 (2019)

[2] Hsu, H.-P., Kremer, K.: Erratum: "a coarse-grained polymer model for studying the glass transition" [j. chem. phys. 150, 091101 (2019)]. J. Chem. Phys. **150**, 159902 (2019)

[3] Kremer, K., Grest, G.S.: Dynamics of entangled linear polymer melts: a molecular-dynamics simulation. J. Chem. Phys. **92**, 5057 (1990)

[4] Kremer, K., Grest, G.S.: Simulations for structural and dynamic properties of dense polymer systems. J. Chem. Soc. Faraday Trans **88**, 1707 (1992)

[5] Bird, R.B., Armstrong, R.C., Hassager, O.: Dynamics of Polymeric Liquids vol. 1 and 2. Wiley, New York (1977)

[6] Doi, M., Edwards, S.F.: The Theory of Polymer Dynamics. Oxford University Press, New York (1986)

[7] Fetters, L.J., Lohse, D.J., Richter, D., Witten, T.A., Zirkel, A.: Connection between polymer molecular weight, density, chain dimensions, and melt viscoelastic properties. Macromolecules **27**, 4639–4647 (1994)

[8] Wischnewski, A., Monkenbusch, M., Willner, L., Richter, D., Likhtman, A.E., McLeish, T.C.B., Farago, B.: Molecular observation of contour-length fluctuations limiting topological confinement in polymer melts. Phys. Rev. Lett. **88**, 058301 (2002)

[9] Wischnewski, A., Richter, D.: Polymer dynamics in melts. In: Gompper, G., Schick, M. (eds.) Soft Matter, Vol. 1:Polymer Melts and Mixtures, pp. 17–85. Wiley-VCH, Weinheim (2006). Chap. 1

[10] Graessley, W.W.: Polymeric Liquids & Networks: Structure and Properties. Garland Science, London and New York (2008)

[11] Herrmann, A., Kresse, B., Wohlfahrt, M., Bauer, I., Privalov, A.F., Kruk, D., Fatkullin, N., Fujara, F., Rössler, E.A.: Mean square displacement and reorientational correlation function in entangled polymer melts revealed by field cycling ^{1}h and ^{2}h nmr relaxometry. Macromolecules **45**, 6516–6526 (2012)

[12] Kremer, K., Grest, G.S., Carmesin, I.: Crossover from rouse to reptation dynamics: A molecular-dynamics simulation. Phys. Rev. Lett. **61**, 566–569 (1988)

[13] Paul, W., Binder, K., Heermann, D.W., Kremer, K.: Dynamiccs of polymer solutions and melts. reptation predictions and scaling of relaxation times. J. Chem. Phys. **95**, 7726 (1991)

[14] Pütz, M., Kremer, K., Grest, G.S.: What is the entanglement length in a polymer melt? Europhys. Lett. **49**, 735–741 (2000)

[15] Harmandaris, V.A., Mavrantzas, V.G., Theodorou, D.N., Kröger, M., Ramirez, J., Öttinger, H.C., Vlassopoulos, D.: Crossover from the rouse to the entangled polymer melt regime: Signals from long, detailed atomistic molecular dynamics simulations, supported by rheological experiments. Macromolecules **36**, 1376–1387 (2003)

[16] Hou, J.-X., Svaneborg, C., Everaers, R., Grest, G.S.: Stress relaxation in entangled polymer melts. Phys. Rev. Lett. **105**, 068301 (2010)

[17] Hsu, H.-P., Kremer, K.: Static and dynamic properties of large polymer melts in equilibrium. J. Chem. Phys. **144**, 154907 (2016)

[18] Hsu, H.-P., Kremer, K.: Detailed analysis of rouse mode and dynamic scattering function of highly entangled polymer melts in equilibrium. Eur. Phys. J. Special Topics **226**, 693 (2017)

[19] Faller, R., Kolb, A., Müller-Plathe, F.: Local chain ordering in amorphous polymer melts: Influence of chain stiffness. Phys. Chem. Chem. Phys. **1**, 2071 (1999)

[20] Faller, R., Müller-Plathe, F., Heuer, A.: Local reorientation dynamics of semiflexible polymers in the melt. Macromolecules **33**, 6602 (2000)

[21] Faller, R., Müller-Plathe, F.: Chain stiffness intensifies the reptation characteristics of polymer dynamics in the melt. Chem. Phys. Chem. **2**, 180 (2001)

[22] Everaers, R., Sukumaran, S.K., Grest, G.S., Svaneborg, C., Sivasubramanian, A., Kremer, K.: Rheology and microscopic topology of entangled polymeric liquids. Science **303**, 823 (2004)

[23] Sukumaran, S.K., Grest, G.S., Kremer, K., Everaers, R.: Identifying the primitive path mesh in entangled polymer liquids. J. Polym. Sci. B **43**, 917 (2005)

[24] Moreira, L.A., Zhang, G., Müller, F., Stuehn, T., Kremer, K.: Direct equilibration and characterization of polymer melts for computer simulations. Macromol. Theor. Simul. **24**, 419 (2015)

[25] Fetters, L.J., Lohse, D.J., Colby, R.H.: Chain dimensions and entanglement spacings. In: Mark, J.E. (ed.) Physical Properties of Polymers Handbook, 2nd, pp. 447–454. Springer, New York (2007). Chap. 25

[26] Hsu, H.-P., Stuehn, T., Daoulas, K.C., Kremer, K.: Molecular dynamics study of the glass transition of highly entangled polymer melts. In: Müller, M., Peter, C., Trautmann, A. (eds.) NIC Symposium 2022 -Proceedings, pp. 135–144. Forschungszentrum Jülich GmbH Zentralbibliothek, Verlag, NIC Series, Jülich, Germany (2022). https://doi.org/http://hdl.handle.net/2128/31840

[27] Hsu, H.-P., Kremer, K.: Efficient equilibration of confined and free-standing films of highly entangled polymer melts. J. Chem. Phys. **153**, 144902 (2020)

[28] Hsu, H.-P., Kremer, K.: Primitive path analysis and stress distribution in highly strained macromolecules. ACS Macro Lett. **7**, 107–111 (2018)

[29] Hsu, H.-P., Kremer, K.: Chain retraction in highly entangled stretched polymer melts. Phys. Rev. Lett. **121**, 167801 (2018)

[30] Hsu, H.-P., Kremer, K.: Clustering of entanglement points in highly strained polymer melts. Macromolecules **52**, 6756–6772 (2019)

[31] Halverson, J.D., Brandes, T., Lenz, O., Arnold, A., Bevc, S., Starchenko, V., Kremer, K., Stuehn, T., Reith, D.: Espresso++: A modern multiscale simulation package for soft matter systems. Comput. Phys. Commun. **184**, 1129–1149 (2013)

[32] Guzman, H.V., Tretyakov, N., Kobayashi, H., Fogarty, A.C., Kreis, K., Krajniak, J., Junghans, C., Kremer, K., Stuehn, T.: Espresso++ 2.0: Advanced methods for multiscale molecular simulations. Comput. Phys. Commun. **238**, 66–76 (2019)

[33] Singh, M.K., Hu, M., Cang, Y., Hsu, H.-P., Therien-Aubin, H., Koynov, K., Fytas, G., Landfester, K., Kremer, K.: Glass transition of disentangled of entangled polyemr melts: single-chain-nanoparticles approach. Macromolecules **53**, 7312–7321 (2020)

[34] Gelb, L.D., Gubbins, E.: Pore size distributions in porous glasses: A computer simulation study. Langmuir **15**, 305–308 (1999)

[35] Thomson, K.T., Gubbins, K.E.: Modeling structural morphology of microporous carbons by reverse monte carlo. Langmuir **16**, 5761–5773 (2000)

[36] Pikunic, J., Clinard, C., Cohaut, N., Gubbins, K.E., Guet, J.-M., Pellenq,

R.J.-M., Rannou, I., Rouzaud, J.-N.: Structural modeling of porous carbons: Constrained reverse monte carlo method. Langmuir **19**, 8565–8582 (2003)

[37] Bhattacharya, S., Gubbins, K.E.: Fast method for computing pore size distributions of model materials. Langmuir **22**, 7726–7731 (2006)

[38] Hansen, J.-P., McDonald, I.R.: Theory of Simple Liquids: with Applications to Soft Matter. Elsevier Science, Amersterdan (2013)

[39] Kumar, S.K., Russell, T.P., Hariharan, A.: Monte carlo simulations of the free surface of polymer melts. Chem. Eng. Sci **49**, 2899–2906 (1994)

[40] Peter, S., Meyer, H., Baschnagel, J.: Thickness-dependent reduction of the glass-transition temperature in thin polymer films with a free surface. J. Polym. Sci.: Part B: Polym. Phys. **44**, 2951–2967 (2006)

[41] Sorichetti, V., Hugouvieux, V., Kob, W.: Determining the mesh size of polymer solutions via the pore size distribution. Macromolecules **53**, 2568–2581 (2020)

[42] Milner, S.T.: The glass transition temperature of polystyrene: Results of a round robin test. J. Therm. Analysis **46**, 965–972 (1996)

[43] Brunauer, S., Emmett, P.H., Teller, E.: Adsorption of gases in multimolecular layers. Journal of the American chemical society **60**(2), 309–319 (1938)

[44] Kargar, F., Balandin, A.A.: Brillouin–mandelstam light-scattering spectroscopy. Nat. Photon. **15**, 720–731 (2021). https://doi.org/10.1038/s41566-021-00836-5

[45] Cheng, W., Sainidou, R., Burgardt, P., Stefanou, N., Kiyanova, A., Efremov, M., Fytas, G., Nealey, P.F.: Elastic properties and glass transition of supported polymer thin films. Macromolecules **40**, 7283–7290 (2007). https://doi.org/10.1021/ma071227i
